# Poor outcomes associated with antithrombotic undertreatment in patients with atrial fibrillation attending Gondar University Hospital: a retrospective cohort study

**DOI:** 10.1186/s12959-018-0177-1

**Published:** 2018-09-18

**Authors:** Eyob Alemayehu Gebreyohannes, Akshaya Srikanth Bhagavathula, Henok Getachew Tegegn

**Affiliations:** Department of Clinical Pharmacy, University of Gondar, Gondar, Ethiopia

**Keywords:** Atrial fibrillation, Antithrombotic, Anticoagulant, Ischemic stroke, Ethiopia

## Abstract

**Background:**

Atrial fibrillation (AF) is a major risk factor for stroke as it increases the incidence of stroke nearly fivefold. Antithrombotic treatment is recommended for the prevention of stroke in AF patients. However, majorly due to fear of risk of bleeding, adherence to recommendations is not observed. The aim of this study was to investigate the impact of antithrombotic undertreatment, on ischemic stroke and/or all-cause mortality in patients with AF.

**Methods:**

A retrospective cohort study was conducted from January 7, 2017 to April 30 2017 using medical records of patients with AF attending Gondar University Hospital (GUH) between November 2012 and September 2016. Patients receiving appropriate antithrombotic management and those on undertreatment, were followed for development of ischemic stroke and/or all-cause mortality. Kaplan-Meier and a log-rank test was used to plot the survival analysis curve. Cox regression was used to determine the predictors of guideline-adherent antithrombotic therapy.

**Results:**

The final analysis included 159 AF patients with a median age of 60 years. Of these, nearly two third (64.78%) of patients were receiving undertreatment for antithrombotic medications. Upon multivariate analysis, history of ischemic stroke/transient ischemic attack (TIA) was associated with lower incidence of antithrombotic undertreatment. A significant increase (HR: 8.194, 95% CI: 2.911–23.066)] in the incidence of ischemic stroke and/or all-cause mortality was observed in patients with undertreatment. Up-on multivariate analysis, only increased age was associated with a statistically significant increase incidence of ischemic stroke and/or all-cause mortality, while only history of ischemic stroke/TIA was associated with a decrease in the risk of ischemic stroke and/or all-cause mortality.

**Conclusion:**

Adherence to antithrombotic guideline recommendations was found to be crucial in reducing the incidence of ischemic stroke and/or all-cause mortality in patients with AF without increasing the risk of bleeding. However, undertreatment to antithrombotic medications was found to be high (64.78%) and was associated with poorer outcomes in terms of ischemic stroke and/or all-cause mortality. Impact on practice: This research highlighted the magnitude of antithrombotic undertreatment and its impact on ischemic stroke and/or all-cause mortality in patients with AF. This article has to alert prescribers to routinely evaluate AF patients’ risk for ischemic stroke and provide appropriate interventions based on guideline recommendations.

## Background

Atrial fibrillation (AF) is one of the most common cardiovascular problems worldwide the prevalence of which has been increasing over the years with an estimated 33.5 million people affected globally [1, 2]. It is a major risk factor for stroke as it increases the incidence of stroke nearly fivefold. It nearly doubles the risk of mortality when compared to non-AF stroke and is associated with increased frequency and functional deficits secondary to ischemic stroke [3]. Its prevalence increases with older age; however, unlike other risk factors of stroke such as hypertension and coronary heart disease, the effect of AF on the risk of stroke doesn’t weaken with advancing age [2, 4].

Earlier studies identified mitral stenosis (MS) as a high risk factor for arterial embolization in patients with AF [5–8] and such patients along with those having mechanical or bioprosthetic heart valves and mitral valve repair have been commonly referred to as having valvular AF [9]. In these patients, a significant reduction in the incidence of systemic embolization has been achieved with oral anticoagulants and withdrawal of anticoagulants has been associated with recurrence of thromboembolic events [6, 10, 11]. Thus, anticoagulation with vitamin K antagonists has been recommended for such patients. As a result these patients have since been generally excluded from studies that evaluated the outcomes of anticoagulation [12–21].

AF patients other than those having “valvular AF” are known to have non-valvular AF (NVAF). As stroke risk among NVAF patients vary, different stroke risk stratification tools including the CHADS_2_ score have been used over the years and currently the CHA_2_DS_2_-VASc score is recommended [9, 22, 23]. NVAF patients can be stratified into low, intermediate, and high risk to stroke depending on whether their CHA_2_DS_2_-VASc scores are 0, 1, or ≥ 2, respectively. A similar categorization may be done using the CHADS_2_ score, however, patients with a CHADS_2_ score of 0 may not all be low risk when stratified using CHA_2_DS_2_-VASc score. Therefore, the CHA_2_DS_2_-VASc has an important advantage of identifying patients who are truly low risk [24].

Oral anticoagulation therapy is the standard management recommended for the prevention of stroke in AF patients with valvular-AF [9, 12] and high risk NVAF patients [9, 22, 23]. However, majorly due to fear of risk of bleeding, adherence to recommendations is not observed and underprescription is now a major barrier to effective anticoagulation. Hence, variability in practice and underutilization of antithrombotic agents as a result of non-adherence to guidelines recommendations can increase the risk of stroke, thromboembolic events, and death [2, 25–29].

The prevalence of cardiovascular diseases including AF in Ethiopia is on the rise. In 2014, cardiovascular diseases were estimated to account to 9% of total deaths in the country [30]. To the best of the authors’ knowledge studies that assessed the impact of undertreatment with antithrombotic agents with AF patients on clinical outcomes are lacking. Therefore, we aimed to measure the adequacy of antithrombotic medication use and to investigate the impact of antithrombotic undertreatment, on ischemic stroke and/or all-cause mortality in patients with AF.

## Methods

### Study setting and period

The study was conducted from January 7, 2017 to April 30 2017 at Gondar University Hospital (GUH). GUH is a teaching and referral hospital located in the northwest Ethiopia 727 k meter from the capital Addis Ababa. The hospital gives service to estimated 7 million people. The medical inpatient ward comprises of 62 beds, 34 beds for males and 28 beds for females.

### Study design

A census using retrospective cohort study was conducted using medical records of patients, 18 years and older, with AF attending the medical inpatient ward and chronic ambulatory clinic of GUH between November 2012 and September 2016. Patients’ medical records were selected based on diagnosis of AF regardless of the presence or absence of other comorbid diseases. The CHA_2_DS_2_-VASc score [9, 31] was calculated to estimate the risk of stroke in patients with NVAF and classify patients into high, moderate, and low risk categories. This score was used to determine the appropriateness of antithrombotic agents. However, as anticoagulation is recommended for all patients with valvular AF, no score was calculated for these patients. Based on this, patients were classified into two groups: guideline adherent treatment vs undertreatment according to the “2016 European Society of Cardiology (ESC) Guidelines for the management of atrial Fibrillation” [9] and the “2014 2014 American Heart Association/American College of Cardiology (AHA/ACC) Guideline for the Management of Patients With Valvular Heart Disease” [12]. The two groups were then followed for occurrence ischemic stroke and/or all-cause mortality. Predictors of ischemic stroke and/or all-cause mortality will then be assessed.

### Statistical analysis

Descriptive statistics were used to summarize sociodemographic and other baseline information. Categorical variables were expressed as frequencies (percentage) and quantitative variables as mean ± standard deviation or median + interquartile range (IQR)/range. Baseline intergroup comparisons were made using a X^2^ test (or a Fisher’s exact test if any expected cell count was < 5) and Pearson’s correlation. Patients in the two groups, i.e. those receiving appropriate antithrombotic management and those on undertreatment, were followed for development of clinical events (ischemic stroke and/or all-cause mortality). Kaplan-Meier and a log-rank test was used to plot the survival analysis curve. A stepwise cox hazard regression was used to determine the predictors of guideline-adherent antithrombotic therapy use including into the model all the candidate variables (variables with *p* ≤ 0.10 in univariate, except those with a high number of missing data). A two-sided statistical tests at 5% level of significance was used. All of the analyses were performed using statistical package for social sciences (SPSS) version 20 (IBM Corp., Armonk, NY).

### Definition of terms and operational definitions

*NVAF*: AF in the absence of rheumatic mitral stenosis, a mechanical or bioprosthetic heart valve, or mitral valve repair; *Paroxysmal AF*: *AF that terminates spontaneously or with intervention within 7 days of onset; Persistent AF:* Continuous AF that is sustained > 7 days; *Longstanding persistent AF:* Continuous AF > 12 months in duration; *Permanent AF:* The term *“permanent AF”* is used when the patient and clinician make a joint decision to stop further attempts to restore and/or maintain sinus rhythm; *Guideline adherent treatment*: prescribing OAC for patients with valvular AF; or prescribing OAC in NVAF patients with CHA2DS2-VASc score ≥ 2; or prescribing an antithrombotic medication (OAC or ASA) in NVAF patients with CHA2DS2-VASc score of 1; or not prescribing any antithrombotic medication in NVAF patients with a CHA2DS2-VASc score of 0; *undertreatment*: prescribing ASA only or not prescribing any antithrombotic medication at all in NVAF patients with CHA2DS2-VASc score ≥ 2;; or not prescribing any antithrombotic agent at all in NVAF patients with CHA2DS2-VASc score of 1; or prescribing ASA only or not prescribing any antithrombotic medication at all in patients with valvular AF.

## Results

### Patients’ characteristics

**Fig. 1:**
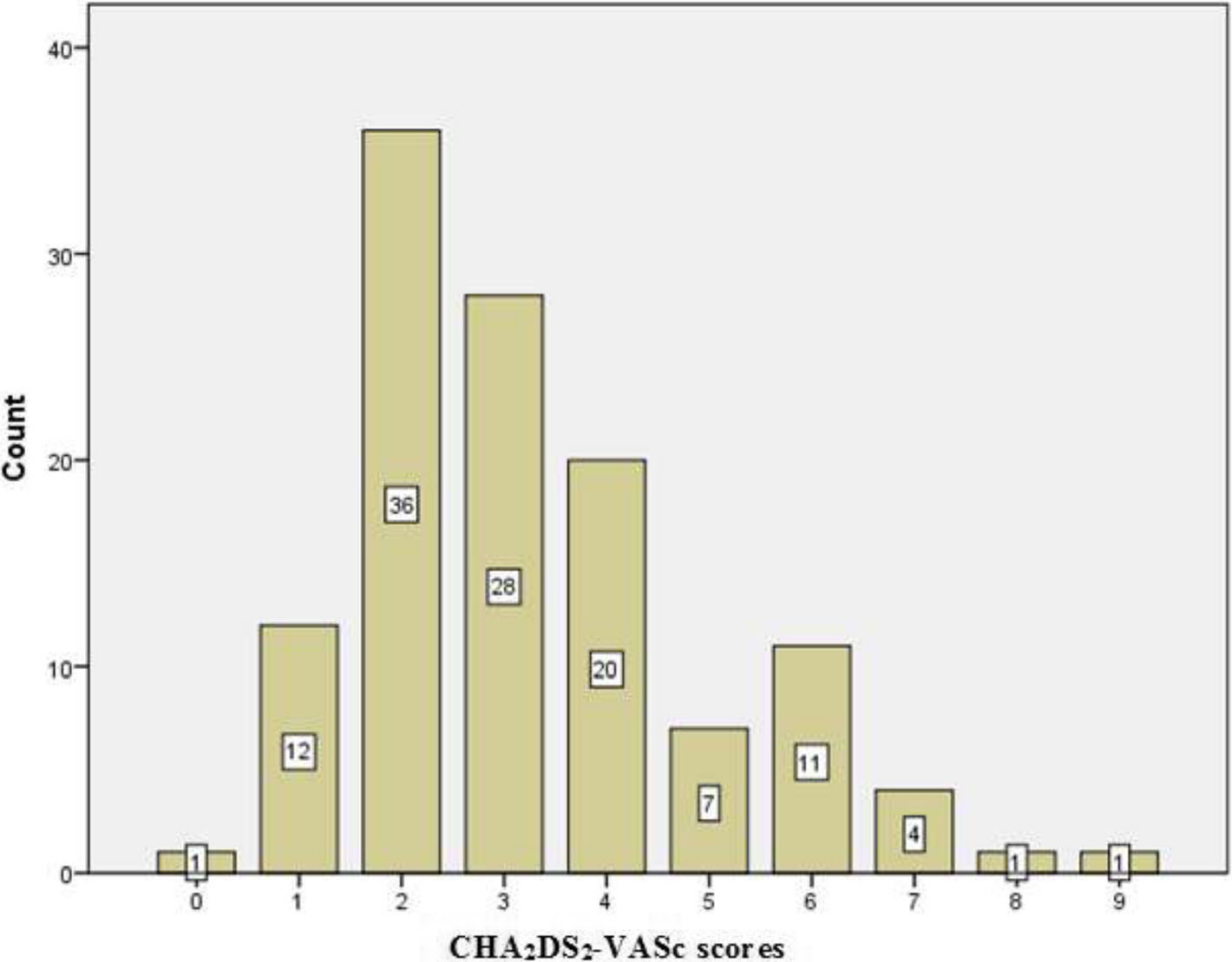
CHA2DS2-VASc scores of patients with NVAF

The study identified a total of 231 patients with AF during the study period. Of these, 72 patients were excluded because either the diagnoses of AF were made once they had developed ischemic stroke with no further follow-up, were with incomplete records, or the medical records were lost from the medical record room. The final analysis included 159 patients with AF. The median (range) age of the patients was 60 (18–90) years with female majority (67.9%). All patients with valvular AF (*N* = 38) have rheumatic MS but none of them had mechanical or bioprosthetic heart valves, or mitral valve repair. On the other hand, patients with NVAF (*N* = 121) had a median CHA_2_DS_2_-VASc score of 3 (range = 0–9) [Fig. 1]. Of these patients, 2 (1.7%), 12 (9.9%), and 108 (89.26%) patients were at low, intermediate, and high risk for the development of ischemic stroke, respectively. Ten (6.3%), 98 (61.6%), 51 (32.1%) patients were having paroxysmal, persistent, and longstanding persistent AF, respectively [Table 1]. For 117 (73.6%) patients, ECG documentation of AF was found.

**Table 1:** Baseline characteristics of AF patients attending GUH, 2017 (*N* = 159)

Variable	All patients *N* = 159	Undertreatment *N* = 103	According to guideline treatment *N* = 56	*P*-value
Age in years				0.09
Mean ± SD	58.50 ± 19.082	62.175 ± 16.215	51.732 ± 22.057	
Median (range)	60 (18–90)	65 (18–89)	51 (18–90)	
IQR	30	18.75	41.75	
Sex				0.989
Males	51 (32.1%)	33 (32.04%)	18 (32.14%)	
Hemoglobin				
Mean ± SD	13.328 ± 2.019	13.226 ± 2.246	13.518 ± 1.574	0.384
Serum creatinine				
Median (IQR)	0.850 (0.32)	0.890 (0.42)	0.765 (0.34)	0.026
SGOT				
Median (IQR)	26.0 (26.95)	27.100 (24.50)	24.55 (38.75)	0.668
SGPT				
Median (IQR)	19.0 (24.3)	18.00 (22.55)	20.00 (33.53)	0.214
AF clinical type				0.003
Valvular AF	38 (23.9%)	17 (16.5%)	21 (37.5%)	
NVAF	121 (76.1%)	86 (83.5%)	35 (62.5%)	
AF pattern				
Paroxysmal	10 (6.3%)	10 (9.7%)	0 (0%)	
Persistent	98 (61.6%)	71 (68.9%)	27 (48.2%)	
Longstanding Persistent	51 (32.1%)	22 (21.4%)	29 (51.8%)	

### Antithrombotic undertreatment/guideline-adherent treatment

One hundred forty five patients, 38 with valvular AF and 107 NVAF patients with CHA_2_DS_2_-VASc score of 2 or more, needed anticoagulation. Twelve of the patients with NVAF also needed at least antiplatelet agents (CHA_2_DS_2_-VASc score of 1). Of these, nearly two third (64.78%) of patients were receiving undertreatment for antithrombotic medications, while the rest were treated according to guideline recommendations. Proportion of antithrombotic undertreatment was higher in patients with NVAF (70.5%) when compared to patients with valvular AF (44.74%).

Compared with those treated according to guideline, patients with undertreatment were but less likely to have a history of ischemic stroke. The two groups have otherwise comparable baseline characteristics. HF and Hypertension were the two most common co-morbidities (Tables 1 and 2) while furosemide and digoxin were the two most commonly prescribed medications in these patients. Of the antithrombotic medications, aspirin (*N* = 66) and warfarin (*N* = 53) were most commonly utilized (Table 3).

**Table 2:** Co-morbidities in patients with AF attending GUH, 2017 (*N* = 159)

	All patients *N* = 159	Potential Undertreatment *N* = 103	According to guideline treatment *N* = 56	*P*-value
CHF	101 (63.5)	63 (61.2%)	38 (67.9%)	0.402
Hypertension	54 (34)	41 (39.8%)	13 (23.2%)	0.080
History of stroke/TIA	30 (18.9)	10 (9.7%)	20 (35.7%)	0.000
Vascular disease	36 (22.6)	21 (20.4%)	15 (26.8%)	0.357
DM	8 (5.0)	6 (5.8%)	2 (3.6%)	0.714
Anemia (Hemoglobin< 12/13)	33 (20.8)	22 (21.4%)	11 (19.64%)	0.799
IHD/ACS	14 (8.8)	10 (9.7%)	4 (7.1%)	0.772
Hyperthyroidism	22 (13.8)	16 (15.5%)	6 (10.7%)	0.400
Cardiomyopathy	5 (3.1)	3 (2.9%)	2 (3.6%)	1.000
Cardiomegaly	43 (27.0)	23 (22.3%)	20 (35.7%)	0.070
Increased LV wall thickness	5 (3.1)	1 (1.0%)	4 (7.1%)	0.052
LVH	18 (11.3)	12 (11.7%)	6 (10.7%)	0.859
LA enlargement	10 (6.3)	6 (5.8%)	4 (7.1%)	0.742
Liver disease (LFT > 3XULN)	11 (6.9%)	6 (5.8%)	5 (8.9%)	0.461
History of bleeding	5 (3.1%)	2 (1.9%)	3 (5.4%)	0.236

**Table 3:** Commonly prescribed medications in patients with AF attending GUH, 2017 (*N* = 159)

	All patients *N* = 159	Potential Undertreatment *N* = 103	According to guideline treatment *N* = 56
ASA	66 (41.5%)	39 (37.9%)	27 (48.2%)
Warfarin	53 (33.3%)	1 (1.0%)	52 (92.9%)
Clopidogrel	5 (3.1%)	3 (2.9%)	2 (3.6%)
Digoxin	74 (46.5%)	43 (41.7%)	30 (53.6%)
Atenolol	58 (36.5%)	29 (28.2%)	29 (51.8%)
Metoprolol	17 (10.7%)	8 (7.8%)	9 (16.1%)
Carvedilol	2 (1.3%)	2 (1.9%)	0 (0%)
Propranolol	10 (6.3%)	8 (7.8%)	2 (3.6%)
Simvastatin	21 (13.2%)	12 (11.7%)	9 (16.1%)
Atorvastatin	20 (12.6%)	10 (9.7%)	10 (17.9%)
Captopril	1 (0.6%)	0 (0%)	1 (1.8%)
Enalapril	33 (20.8%)	17 (16.5%)	16 (28.6%)
Furosemide	89 (56.0%)	53 (51.5%)	36 (64.3%)
Spironolactone	72 (45.3%)	43 (41.7%)	29 (51.8%)
Hydrochlorothiazide	14 (8.8%)	9 (8.7%)	5 (8.9%)
Nifedipine/amlodipine	3 (1.9%)	2 (1.9%)	1 (1.8%)
Monthly benzathine penicillin	17 (10.7%)	3 (2.9%)	14 (25.0%)
PTU	19 (11.9%)	12 (11.7%)	7 (12.5%)

### Predictors of antithrombotic undertreatment in patients with AF

Valvular AF, older age, hypertension, history of stroke/TIA, higher serum creatinine, VHD, and medications such as atenolol and monthly benzathine penicillin were identified in a univariate analysis as factors that decrease in the incidence antithrombotic undertreatment. However, upon multivariate analysis, only history of ischemic stroke/TIA and prescription of atenolol and enalapril were associated with lower incidence of antithrombotic undertreatment (Table 4).

**Table 4:** Predictors of antithrombotic undertreatment among AF patients attending GUH, 2017 (*N* = 159)

Variables		COR (95% CI)	p-value	AOR (95% CI)	*p*-value
Clinical type	Valvular AF	0.329 (0.156–0.698)	0.004	1.116 (0.285–4.367)	0.875
Age	in years	1.030 (1.011–1.048)	0.001	1.009 (0.979–1.040)	0.575
Hypertension	Yes	2.187 (1.049–4.562)	0.037	2.849 (0.926–8.770)	0.068
History of stroke/TIA	Yes	0.194 (0.083–0.453)	0.000	0.054 (0.017–0.175)	0.000
Serum creatinine	mg/dL	3.328 (1.135–9.753)	0.028	1.901 (0.582–6.208)	0.287
Cardiomegaly	Yes	0.518 (0.253–1.060)	0.072	0.657 (0.239–1.807)	0.416
Increased LV wall thickness	Yes	0.127 (0.014–1.170)	0.069	0.135 (0.003–5.344)	0.286
ECG documentation	Yes	0.507 (0.230–1.121)	0.093	0.351 (0.120–1.029)	0.056
VHD	Yes	0.377 (0.188–0.757)	0.006	0.392 (0.149–1.036)	0.059
Atenolol	Yes	0.365 (0.185–0.718)	0.004	0.362 (0.144–0.910)	0.031
Enalapril	Yes	0.494 (0.227–1.077)	0.076	0.317 (0.107–0.942)	0.039
Monthly benzathine penicillin	Yes	0.090 (0.025–0.330)	0.000	0.186 (0.030–1.167)	0.073

### Survival analysis

**Fig. 2:**
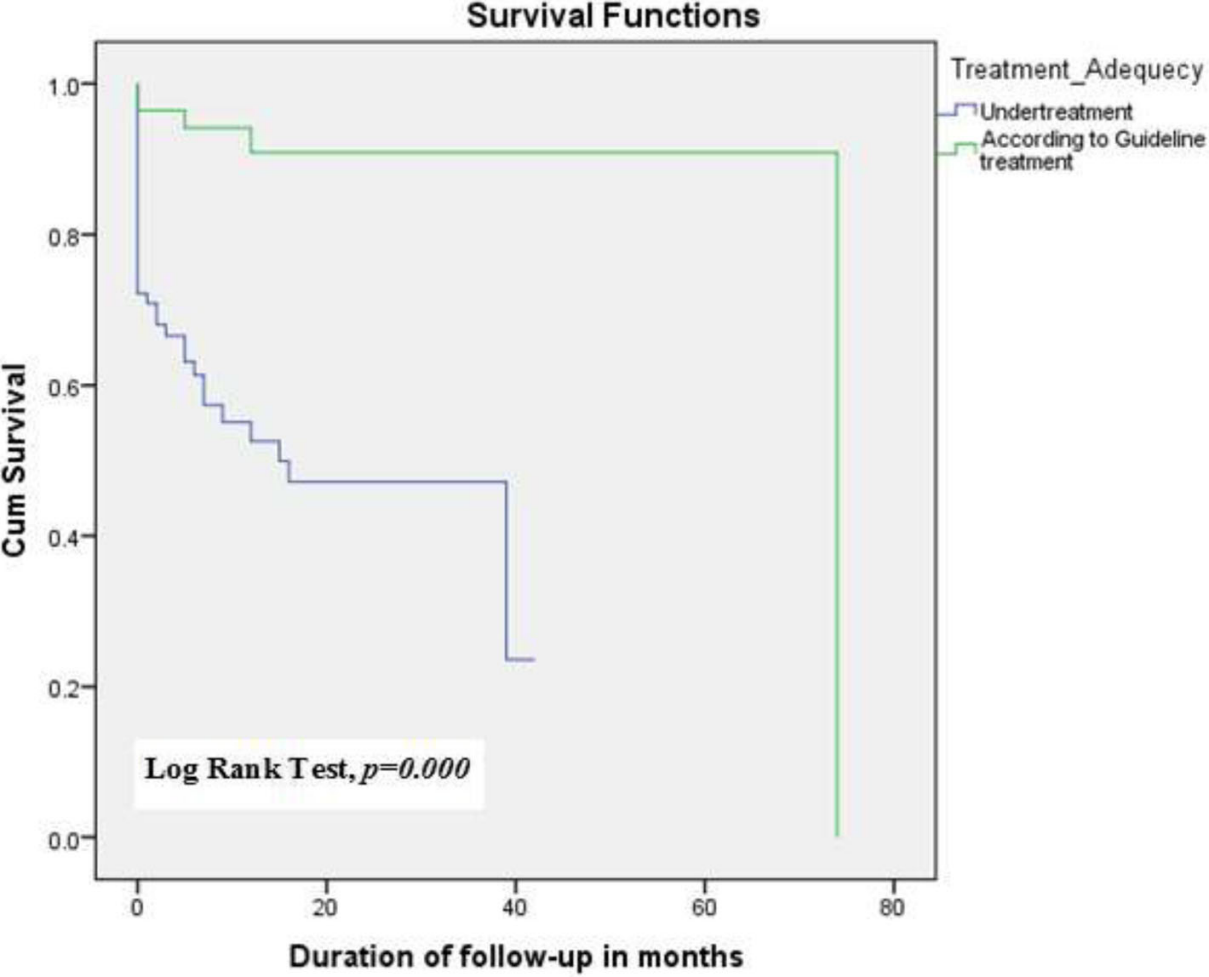
Kaplan-Meier survival analysis curve in patients with potential antithrombotic undertreatment and guideline-adherent treatment

The median duration of follow-up was 15.00 months for undertreatment group and 74.00 months for according to guideline treatment, respectively. During the follow-up period, a total of 52 (32.7%) patients developed ischemic stroke, 47 patients from the undertreatment group and 5 from the guideline-adherent group. Five cases of bleeding were reported but there was no statistically significant difference between the two groups (*p* = 0.980). Eight patients died during the follow-up period 7 of which were receiving undertreatment. Kaplan-Meier (log Rank test, *p* = 0.000) and Cox regression analyses (AHR: 8.194, 95% CI: 2.911–23.066) showed a significant increase in the incidence of ischemic stroke and/or all-cause mortality in patients with undertreatment [Fig. 2]. A sub-group analysis of patients with NVAF also revealed a similar result (AHR: 7.511, 95% CI: 2.295–24.580, *p* = 0.001).

### Predictors of ischemic stroke and/or all-cause mortality in patients with AF

Up-on univariate analysis, cox proportional hazard regression revealed that NVAF, older age, hypertension were associated with higher risk of ischemic stroke and/or all-cause mortality. On the other hand, the presence of CHF, history of ischemic stroke/TIA, cardiomegaly, presence of any type of valvular disease, and use of medications such as ASA, warfarin, digoxin, atenolol, enalapril, furosemide and spironolactone were associated with a decrease in the risk of ischemic stroke and/or all-cause mortality. However, up-on multivariate analysis, only increased age was associated with a modest but statistically significant increase risk for ischemic stroke and/or all-cause mortality (AHR: 1.035, 95% CI = 1.004–1.067), while only history of ischemic stroke/TIA was associated with a decrease in the risk of ischemic stroke and/or all-cause mortality (AHR: 0.038, 95% CI: 0.002–0.596) (Table 5).

**Table 5:** Predictors of ischemic stroke and/or all-cause mortality in patients with AF attending GUH, 2017 (*N* = 159)

Variables		CHR (95% CI)	*p*-value	AHR (95% CI)	*p*-value
Clinical type	NVAF	3.374 (1.321–8.614)	0.011	0.784 (0.194–3.167)	0.733
Age	in years	1.039 (1.019–1.060)	0.000	1.035 (1.004–1.067)	0.029
CHF	Yes	0.223 (0.119–0.416)	0.000	0.347 (0.105–1.141)	0.081
Hypertension	Yes	1.970 (1.097–3.536)	0.023	1.117 (0.410–3.042)	0.828
History of stroke/TIA	Yes	0.371 (0.133–1.036)	0.058	0.038 (0.002–0.596)	0.020
Cardiomegaly	Yes	0.484 (0.225–1.040)	0.063	0.843 (0.316–2.244)	0.732
LVEF	in %	1.033 (1.006–1.061)	0.017	1.051 (0.998–1.106)	0.059
Any type of Valvular disease	Yes	0.429 (0.234–0.788)	0.006	1.757 (0.660–4.679)	0.259
AF pattern					
Paroxysmal		–	–	–	–
Persistent		0.361 (0.158–0.826)	0.016	1.293 (0.287–5.830)	0.738
Longstanding persistent		0.016 (0.003–0.074)	0.000	0.002 (0.000–1.875)	0.075
ASA	Yes	0.443 (0.231–0.850)	0.014	1.179 (0.256–5.426)	0.832
Warfarin	Yes	0.099 (0.030–0.320)	0.000	0.294 (0.033–2.642)	0.275
Digoxin	Yes	0.300 (0.151–0.597)	0.001	1.259 (0.678–2.337)	0.465
Atenolol	Yes	0.146 (0.057–0.373)	0.000	0.139 (0.017–1.125)	0.064
Enalapril	Yes	0.364 (0.143–0.926)	0.034	1.395 (0.087–22.248)	0.814
Furosemide	Yes	0.201 (0.101–0.397)	0.000	0.416 (0.057–3.008)	0.385
Spironolactone	Yes	0.149 (0.063–0.354)	0.000	0.121 (0.013–1.134)	0.064

## Discussion

This retrospective analysis of medical records of AF patients assessed adequacy of antithrombotic treatment using 2016 ESC [7] and 2014 AHA/ACC [8] guidelines to evaluate outcomes of undertreatment. The findings of this study showed that adherence to guideline recommendations was associated with significantly better outcomes. Incidence of the primary endpoint (ischemic stroke and/or all-cause mortality) was increased by more than eight-folds (AHR: 8.194, 95% CI: 2.911–23.066) in patients with antithrombotic undertreatment. A statistically significant (*p* = 0.000) difference in the duration of follow-up between patients with guideline-adherent treatment (median: 74 months) and undertreatment (median: 15 months) was also observed up-on Kaplan-Meier analysis.

These observations stress the urgent need of effective antithrombotic treatment by practicing adherence to 2016 ESC [7] and 2014 AHA/ACC [8] guideline recommendations. Prevention of ischemic stroke should be an integral part in the management of patients with AF and clinicians should routinely evaluate their patients for risk of ischemic stroke. NVAF patients with CHA_2_DS_2_-VASc score of 2 or more and all patients with valvular AF are particularly at high risk for the development of ischemic stroke and as such should be provided with oral anticoagulant medications.

Earlier studies identified a substantial increase in the incidence of ischemic stroke in patients with valvular AF that was shown to be significantly decreased with the use of oral anticoagulants, particularly vitamin k antagonists, and recurrence of thromboembolic events was observed upon withdrawal of anticoagulants [5, 8, 10, 11]. On the other hand, CHA_2_DS_2_-VASc score has proven useful in the management of patients with NVAF. Lip et al. reported that guideline non-adherence was associated with an increase in the incidence of ischemic stroke and thromboembolic events (AHR: 1.679, 95% CI: 1.202–2.347) [32] in patients with NVAF. Similarly, the CHA_2_DS_2_-VASc score was also found very important in the current study stratifying patients with NVAF into different risk categories. Accordingly, non-adherence to antithrombotic guideline recommendations was associated with an enormous increase in the incidence of ischemic stroke and/or all-cause mortality (AHR: 7.511, 95% CI: 2.295–24.580).

The findings of this study showed that undertreatment of antithrombotic medications was very high (64.78%). This was much higher than that was reported by Lip et al. (17.3%) [32]. Proportion of undertreatment was particularly higher (70.5%) in patients with NVAF. On the other hand, Basaran et al. reported a 30.5% rate of antithrombotic undertreatment in patients with NVAF [33] which is much lower than the present study. Fear of bleeding and underestimation of the benefit of antithrombotic treatment have been mentioned as major reasons for antithrombotic undertreatment [9, 30, 34]. In particular, fear of bleeding might be the main reason for the observed high proportion of undertreatment in our study; however, our study didn’t assess reasons for this undertreatment.

Predictors of ischemic stroke and/or all-cause mortality and guideline non-adherence were also assessed in this study. On a multivariate analysis, only older age was associated with a statistically significant increase in the incidence of ischemic stroke and/or all-cause mortality upon cox regression [AHR (95% CI): 1.035 (1.004–1.067), *p* = 0.029]. In other studies, history of ischemic stroke, older age, vascular disease, diabetes, female gender, and hypertension were identified as predictors of ischemic stroke and/or thromboembolic events on multivariate analyses [28, 35]. Similar to the Firberg et al. study [35], heart failure and thyroid disease were not identified as predictors of ischemic stroke and/or all-cause mortality in the present study. Older age, female sex, first detected and paroxysmal AF have been identified as predictors of poor adherence to guidelines in other studies [28, 32, 36]. However, none of these factors were identified as predictors of adherence to guideline recommendations in the present study. On the other hand, history of ischemic stroke/TIA was associated with at lower incidence of ischemic stroke in the present study. This might be explained by the fact that, physicians’ tendency to prescribe antithrombotic medications once patients develop ischemic stroke/TIA with thinking the risk of developing ischemic stroke outweighs any potential adverse event especially the risk of bleeding. This justification was supported by the fact that history of stroke/TIA was associated with lower incidence of undertreatment in our study. Mochalina et al. also identified history of ischemic stroke as a factor that increase the odds of oral anticoagulant prescription in patients with NVAF [28]. He also reported that oral anticoagulant use didn’t strictly follow stroke risk assessment as only three (history of ischemic stroke, hypertension, and older age) of the seven risk factors in the CHA_2_DS_2_-VASc score were associated with increased odds of oral anticoagulant medication use. In the present study, in addition to history of ischemic stroke, use of medications such as atenolol [AHR (95% CI): 0.362 (0.144–0.910), *p* = 0.031] and enalapril [AHR (95% CI): 0.317 (0.107–0.942), *p* = 0.039] was also associated with better guideline adherence.

Five patients (3.14%) experienced bleeding. Of these, one patient experienced GIB while on ASA. Four patients experienced epistaxis and/or blood in sputum of which 3 patients were receiving both ASA and warfarin while the remaining patient was receiving ASA. 5.03% (*N* = 8) of the study participants died. Of these, 1 patient was with valvular AF while the remaining 7 patients were with NVAF. This gave us an all-cause mortality rate of 5.79% in patients with NVAF.

In our study, warfarin was the only oral anticoagulant used by any of the patients. A number of novel oral anticoagulants are now currently in use world-wide. Several studies that compared these medications indicated that this medications have at least comparable efficacy with more or less similar, if not better, safety profile in terms of bleeding and mortality particularly in patients with NVAF [37–39]. In addition, they showed better persistence than warfarin [40]. These advantages makes the novel oral anticoagulants alternatives to these patients especially those with NVAF as they haven’t extensively studied in patients with valvular AF. These medications were also suggested to be cost-effective in terms of life-years gained and quality-adjusted life years in developed countries [41, 42], however, this might not be the case in developing countries like Ethiopia as the cost-effectiveness studies were based on willingness to pay which definitely will not be the same depending on the income status of the countries.

### Study limitations

Though the study clearly assessed adequacy of antithrombotic treatment and outcomes of undertreatment, it is not without limitations. The sample size was small which may obscure the impact of some predictors that would have been evident with a larger sample size. It was a retrospective study design and suffered from incompleteness and even loss of patients’ medical records. The study also didn’t assess the bleeding risk of patients. Therefore, interpretation of the results of these study should be in light of these limitations.

## Conclusion

Adherence to 2016 ESC and 2014 AHA/ACC antithrombotic guideline recommendations was found to be crucial in reducing the incidence of ischemic stroke and/or all-cause mortality in patients with AF without increasing the risk of bleeding. However, undertreatment to antithrombotic medications was found to be high and was associated with poorer outcomes in terms of composite end points of thromboembolic events and/or. Even if increased age was associated with a statistically significant increase risk for ischemic stroke and/or all-cause mortality, it was very modest. On the other hand, a tendency to prescribe antithrombotic medications in AF patients with a history of ischemic stroke/TIA was observed and was associated with a decrease in the risk of composite end points of stroke and/or mortality as well as undertreatment.

## Abbreviations

ACC: American College of Cardiology
ACS: Acute Coronary Syndrome
AF: Atrial Fibrillation
AHA: American Heart Association
ASA: Aspirin
DM: Diabetes Mellitus
ESC: European Society of Cardiology
GUH: Gondar University Hospital
HF: Heart Failure
HR: Hazard Ratio
IHD: Ischemic Heart Disease
IQR: Interquartile Range
LA: Left Atrium
LVEF: Left Ventricular Ejection Fraction
LVH: Left Ventricular Hypertrophy
MS: Mitral Stenosis
NVAF: Non-valvular Atrial Fibrillation
OAC: Oral Anticoagulant
OR: Odds Ratio
SD: Standard Deviation
SPSS: Statistical Package for Social Sciences
TIA: Transient Ischemic Attack
ULN: Upper Limit of the Normal
VHD: Valvular Heart Disease
